# Supplementary material for: The estimated hepatitis C seroprevalence and key population sizes in San Diego in 2018
Source: PLoS One. 2021 Jun 9;16(6):e0251635. doi: 10.1371/journal.pone.0251635 (PMC8189442; doi:10.1371/journal.pone.0251635)
Supplement: S1 Text — (DOCX) [file pone.0251635.s001.docx]

**S1 Text. Literature review search strategy.**

The specific search terms for the literature review for each database are listed below.

**Pub Med**

((("incidence"[MeSH Terms] OR "cross-sectional studies"[MeSH Terms] OR "prevalence"[MeSH Terms] OR "cohort studies"[MeSH Terms] OR "epidemiology"[MeSH Terms]) OR (incidence[tw] OR prevalence[tw] OR cross-sectional studies[tw] OR cohort[tw] OR (epidemiolog[tw] OR epidemiologal[tw] OR epidemiologcal[tw] OR epidemiologcial[tw] OR epidemiologi[tw] OR epidemiologia[tw] OR epidemiologiai[tw] OR epidemiologial[tw] OR epidemiologic[tw] OR epidemiologic'[tw] OR epidemiologica[tw] OR epidemiological[tw] OR epidemiological'[tw] OR epidemiologicala[tw] OR epidemiologicaland[tw] OR epidemiologicalcaries[tw] OR epidemiologicalind[tw] OR epidemiologically[tw] OR epidemiologically'[tw] OR epidemiologicalm[tw] OR epidemiologicals[tw] OR epidemiologicalstudies[tw] OR epidemiologicaly[tw] OR epidemiologicas[tw] OR epidemiologicheskie[tw] OR epidemiologici[tw] OR epidemiologicl[tw] OR epidemiologico[tw] OR epidemiologicopedics[tw] OR epidemiologicos[tw] OR epidemiologics[tw] OR epidemiologicy[tw] OR epidemiologiczna[tw] OR epidemiologicznymi[tw] OR epidemiologie[tw] OR epidemiologiecal[tw] OR epidemiologies[tw] OR epidemiologies'[tw] OR epidemiologii[tw] OR epidemiologija[tw] OR epidemiologiocal[tw] OR epidemiologiologic[tw] OR epidemiologique[tw] OR epidemiologiques[tw] OR epidemiologische[tw] OR epidemiologischen[tw] OR epidemiologischer[tw] OR epidemiologising[tw] OR epidemiologising'[tw] OR epidemiologist[tw] OR epidemiologist'[tw] OR epidemiologist's[tw] OR epidemiologists[tw] OR epidemiologists'[tw] OR epidemiologists'estimations[tw] OR epidemiologival[tw] OR epidemiologiy[tw] OR epidemiologle[tw] OR epidemiologos[tw] OR epidemiologucal[tw] OR epidemiology[tw] OR epidemiology'[tw] OR epidemiology's[tw] OR epidemiologya[tw] OR epidemiologyand[tw] OR epidemiologyc[tw] OR epidemiologycal[tw] OR epidemiologyf[tw] OR epidemiologyic[tw] OR epidemiologyical[tw] OR epidemiologymanagement[tw] OR epidemiologyof[tw] OR epidemiologysbt[tw] OR epidemiologywork[tw]))) AND ("hepatitis C"[mesh] OR "hepatitis c"[tw] OR "hep c"[tw] OR HCV[tw] OR anti-hcv[tw] OR hcv-rna[tw])) AND ("san diego"[tw] OR "california"[MeSH Terms] OR california[tw])

**Web of Science**

TS=(incidence OR prevalence OR "cross-sectional studies" OR cohort OR epidemiolog*)

AND

TI=("hepatitis c" OR "hep C" OR hcv OR anti-hcv OR hcv-rna)

AND

ALL=("san diego" OR california)

**Embase**

'incidence'/mj OR 'prevalence'/mj OR 'cross-sectional study'/mj OR 'cohort analysis'/mj OR 'health survey'/mj OR 'seroepidemiology'/mj OR 'incidence':ti,ab OR 'prevalence':ti,ab OR 'cross-sectional study':ti,ab OR 'cohort':ti,ab OR 'epidemiolog*':ti,ab

AND

'hepatitis c'/mj OR 'hepatitis c virus'/mj OR 'hepatitis c':ti,ab OR 'hep c':ti,ab OR 'hcv':ti,ab OR 'anti-hcv':ti,ab OR 'hcv-rna':ti,ab

AND

'california'/mj OR 'california':ti,ab OR 'diego':ti,ab
