# Supplementary figures and images for: The estimated hepatitis C seroprevalence and key population sizes in San Diego in 2018
Source: PLoS One. 2021 Jun 9;16(6):e0251635. doi: 10.1371/journal.pone.0251635 (PMC8189442; doi:10.1371/journal.pone.0251635)

**S1 Fig. Flow diagram for the review**


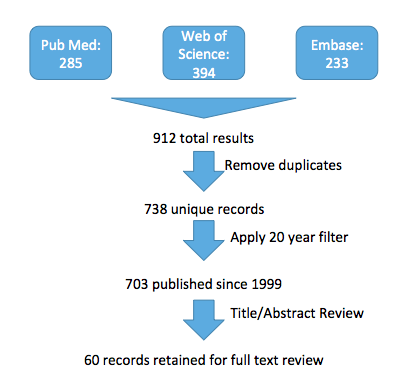

Supplement: S1 Fig — (DOCX) [file pone.0251635.s002.docx]
