# Supplementary material for: The estimated hepatitis C seroprevalence and key population sizes in San Diego in 2018
Source: PLoS One. 2021 Jun 9;16(6):e0251635. doi: 10.1371/journal.pone.0251635 (PMC8189442; doi:10.1371/journal.pone.0251635)
Supplement: S1 Table — (DOCX) [file pone.0251635.s003.docx]

**S1 Table: Results from a sensitivity analysis applying the 2007 PWID prevalence rate of 1.24% (min: 0.19%, max: 2.46%) to the 2018 San Diego County adult population**

| **Subpopulation** |  | **Population Size Point Estimate** | **95% Confidence Interval** | | **HCV seroprevalence (anti-HCV) Point Estimate** | **95% Confidence Interval** | | **# HCV seropositive** | **95% Confidence Interval** | |
| --- | --- | --- | --- | --- | --- | --- | --- | --- | --- | --- |
| PWID |  | 33,915 | 11,344 | 57,528 | 0.6560 | 0.6140 | 0.6942 | 22,270 | 7,412 | 37,906 |
|  |  |  |  |  |  |  |  |  |  |  |
| MSM |  | 88,763 | 61,559 | 120,549 |  |  |  | 4,086 | 2,004 | 6,940 |
|  | HIV positive | 17,038 | 9,063 | 27,565 | 0.1654 | 0.1551 | 0.1759 | 2,818 | 1,494 | 4,578 |
|  | HIV negative (18-54) | 49,767 | 36,552 | 64,273 | 0.0072 | 0.0032 | 0.0118 | 359 | 149 | 649 |
|  | HIV negative (55-74) | 17,479 | 12,722 | 22,811 | 0.0470 | 0.0242 | 0.0808 | 846 | 352 | 1,527 |
|  | HIV negative (75+) | 4,479 | 3,222 | 5,900 | 0.0128 | 0.0018 | 0.0382 | 63 | 9 | 186 |
|  |  |  |  |  |  |  |  |  |  |  |
| General Population (excluding other groups)^bcd^ | | |  |  |  |  |  |  |  |  |
|  | Men 18-54 | 830,421 | 794,290 | 866,766 | 0.0072 | 0.0032 | 0.0118 | 5,979 | 2,542 | 10,228 |
|  | Men 55-74 | 289,319 | 266,724 | 310,647 | 0.0470 | 0.0242 | 0.0808 | 13,598 | 6,455 | 25,100 |
|  | Men 75+ | 76,467 | 69,663 | 83,391 | 0.0128 | 0.0018 | 0.0382 | 979 | 125 | 3,186 |
|  | Women 18-54 | 837,657 | 815,457 | 859,806 | 0.0037 | 0.0018 | 0.0065 | 3,099 | 1,468 | 5,589 |
|  | Women 55-74 | 344,537 | 323,120 | 365,980 | 0.0240 | 0.0122 | 0.0427 | 8,269 | 3,942 | 15,627 |
|  | Women 75+ | 115,507 | 106,928 | 124,078 | 0.0000 | 0.0000 | 0.0000 | 0 | 0 | 0 |
|  | Total general pop | 2,493,908 | 2,376,182 | 2,610,668 |  |  |  | 31,924 | 14,532 | 59,730 |
|  |  |  |  |  |  |  |  |  |  |  |
| People incarcerated in California state prisons^a^ |  | 8793 |  |  | 0.2295 | 0.2218 | 0.2372 | 2,018 | 1,950 | 2,086 |
|  |  |  |  |  |  |  |  |  |  |  |
| TOTAL |  |  |  |  |  |  |  | 60,298 | 25,966 | 106,594 |

Notes: ^a^Individuals incarcerated in 12/31/18 in California with San Diego as their county of commitment. ^b^Excluding other risk populations above. ^c^Blood donor data adjusted by an inflation factor of 4.9 (CI 2.2-7.7) for ‘healthy donor effect’ as per Facente et al. 2018. ^d^Closest age groups to the aged 55-74 1945-1965 in 2018 based on ACS age groupings
